# Relationships of coping styles and psychological distress among patients with insomnia disorder

**DOI:** 10.1186/s12888-021-03254-7

**Published:** 2021-05-17

**Authors:** Yinghui Li, Xiaoyin Cong, Suzhen Chen, Yong Li

**Affiliations:** 1 Department of Psychosomatics and Psychiatry, ZhongDa Hospital, School of Medicine, Southeast University, Nanjing, 210009 China; 2 Department of Clinical Psychology, Jiangsu Province Hospital, Nanjing, 210029 China

**Keywords:** Insomnia, Coping style, Psychological factors, Depression

## Abstract

**Background:**

Insomnia appears to be one of the most frequent sleep complaints in the general population. It has significant negative impact on daily functioning. However, there has been little research that described the effect of coping style in insomnia disorder.

**Methods:**

The Simplified Coping Style Questionnaire (SCSQ) was used to evaluate 79 adult patients with insomnia disorder alongside 80 healthy controls. Additionally, sleep quality was assessed with the Pittsburgh Sleep Quality Index (PSQI), and Symptom Checklist-90-Revised (SCL-90R) was utilized to determine the status of depression, anxiety and other psychological symptoms.

**Results:**

Positive coping style score was significantly lower, whereas negative coping style score and nine symptomatic dimensions of SCL-90R were significantly higher in insomnia patients than in controls. Positive coping style score was adversely related to PSQI score, obsessive-compulsive, depression, anxiety and phobic anxiety, whereas negative coping style score was positively related to PSQI score, somatization and interpersonal sensitivity. Further multiple stepwise regression analysis showed that PSQI total score was independently and positively correlated with negative coping style score.

**Conclusions:**

Insomniacs use more negative coping styles and less positive ones. Positive coping is adversely associated with insomnia symptoms and psychological distress, whereas negative coping is positively related to those symptoms. And negative coping has a negative effect on sleep quality. we should attach importance to coping styles of insomniacs in clinical practice, which may help to develop more targeted prevention and intervention strategies.

## Background

Insomnia, which is described as difficulty initiating and maintaining sleep and/or early morning awakenings, appears to be the most common sleep disorder in the general population [1]. Approximately 10–30% of the population worldwide suffer from insomnia [2]. It has been shown that insomnia is associated with considerable deterioration of quality of life, higher prevalence of depressive disorder, cognitive impairment and higher risk of work absenteeism [3–5]. In addition, insomnia is generally associated with a variety of medical conditions, such as diabetes, heart disease, migraine and cancer [6–9], even higher risk of mortality [10]. Despite the high incidence and negative impact on the individual, insomnia remains an under-recognized condition [11].

The research of coping styles is valued in psychosomatic medicine. Coping styles have been shown to be a stable psychological and behavioral strategy, positive or negative to overcome external and internal challenges [12]. Positive coping style tends to take direct and rational ways to solve problems, in contrast, negative coping style refers to dealing with problems by neglecting, avoidance and denial [13, 14]. Previous studies have demonstrated that negative coping style could increase the likelihood of suicidal ideation when confronted with stressful events, and positive coping style could alleviate depressed mood [13, 15]. Although we know that coping may play a mediating role in the pathogenesis of insomnia [16–18], However, to our knowledge, there has been little research that described the effect of coping style in insomnia disorder. Previous studies on the relationship between insomnia and coping style have mostly been conducted in healthy population or patients with physical diseases [17, 19–21]. A nationwide survey among general Japanese population revealed that distraction-based stress coping such as hobbies and optimistic thinking, was positively associated with sleep disturbance [22]. A cross-sectional study assessing 434 colorectal cancer patients found that positive coping predicted better sleep, whereas higher level of negative coping was related to greater severity in preoperative insomnia symptoms [20]. Another multicenter study was conducted in 404 patients with digestive tract cancer showed that patients’ psychological status suffered significant deterioration and the most commonly used coping style was avoidance after surgery [21].

We hypothesized that patients with insomnia disorder are more likely to use negative coping strategies and less likely to use positive coping strategies, which is closely related to the severity of insomnia and the psychological status. Therefore, we investigated the coping styles in patients with insomnia disorder and examined the association between sleep disturbance or psychological distress and coping styles. In the present study, we used an integrated questionnaire consisting of the Pittsburgh Sleep Quality Index (PSQI), the Symptom Checklist-90-Revised (SCL-90R), and Simplified Coping Style Questionnaire (SCSQ) to assess insomnia severity, psychological distress and coping styles in patients meeting DSM-5 criteria for insomnia disorder.

## Methods

### Participants and setting

Adult outpatients were recruited from the psychological departments of First Affiliated Hospital of Nanjing Medical University in Jiangsu province of China between May 2019 and December 2019. Eligible participants met Diagnostic and Statistical Manual of Mental Disorders, Fifth Edition (DSM-5) diagnostic criteria for chronic insomnia disorder [23]. Adult healthy volunteers were recruited from the Health Management Centre of this hospital as healthy controls. All patients and controls underwent detailed history taking. All subjects with the following conditions were excluded: (a) irregular sleeping patterns, (b) history of diagnosis of alcohol or substance abuse/dependence in the last 6 months, (c) use of antidepressants or antipsychotics, (d) pregnancy for women, (e) other mental disorders, (f) serious physical illness, (g) physical or psychological impairments that prevented from completing the questionnaires. The participants were assessed to determine their sleep quality, coping style, and psychological distress using interviewer-assisted and self-report methods.

### Sociodemographics and lifestyle data

Demographic data including age, gender, marital status, body mass index (BMI), and years of education were recorded in all insomnia patients and healthy controls. Simultaneously, lifestyle data, namely smoking and alcohol consumption were also investigated. Tobacco and alcohol use was classified into regular (more than once a week) and never/rare use. In addition, the course of illness of patients was recorded.

### Sleep quality assessment (PSQI)

Sleep quality was assessed by a Chinese version of PSQI questionnaire which was widely used and validated in Chinese population [24] . It consists of 19 self-rating items and 7 dimensions that measure the quality of sleep: subjective sleep quality, sleep duration, sleep latency, sleep disturbance, habitual sleep efficiency, daytime dysfunction, and used sleep medication. Each dimension is rated from 0 to 3. The sum of the 7 dimension scores gives the global PSQI score, ranging from 0 to 21. Higher scores indicate worse sleep quality.

### Assessment of coping styles

The Simplified Coping Style Questionnaire (SCSQ) is a 20-item self-report scale that measures individual coping style. The SCSQ was divided into two subscales: positive coping (12 items) and negative coping (8 items). Positive coping reflects the level of the active coping style, such as “solving problems by work, learning or other things” or “looking at the good side of things”. In contrast, negative coping reflects the level of passive coping style, such as “when facing problems, escaping troubles by drinking and smoking” or “relying others to solve problems”. Each item is scored on a four-point Likert scale (0 = never, 1 = seldom, 2 = often, 3 = always). Higher scores on each subscale reflect the level of the coping style. Cronbach’s α for positive coping and negative coping were 0.89 and 0.78, respectively [25].

### Evaluation of psychiatric distress

The Chinese version of SCL-90R was used to evaluated the psychiatric distress in patients with insomnia disorder. It is composed of 90 items rated on a Likert-type scale ranging from 1 (no problem) to 5 (very serious), and yields 9 subscales measuring somatization, obsessive-compulsive, interpersonal sensitivity, depression, anxiety, hostility, phobic anxiety, paranoid ideation, and psychoticism. Additionally, there were 7 items that were not included in any subscales were taken as additional items in the analysis [26, 27]. Higher scores indicate more serious psychiatric distress [28]. The Chinese-version SCL-90R has been proved to possess adequate validations and reliability in clinical research [29].

### Data analysis

SPSS version 22.0 (SPSS, Inc., Chicago, IL, USA) statistical software was used for all data analyses. Measurement data are mean ± standard deviation (SD). Various sociodemographic variables, coping style parameters and psychological factors between insomnia patients and healthy controls were assessed by independent sample *t* test. For categorical variables, the chi-square test was used for comparisons between the two groups. The correlations between variables were assessed by Spearman correlation analysis. Multiple stepwise regression analysis, with gender, age, BMI, years of education, duration of symptoms, ten symptom dimensions of SCL-90R, positive coping style score and negative coping style score entered, was applied to explore the independent influencing factors with significant contribution to sleep quality in patients with insomnia disorder. A two-tailed *P* value < 0.05 was considered statistically significant.

## Results

### Sample characteristics

Of the 158 subjects eligible for inclusion, a total of 129 (81.6%) individuals agreed to participate in the study. However, 50 individuals did not complete the questionnaires because of low literacy or voluntary withdrawal. Eventually, a total of 79 insomnia outpatients completed questionnaires, yielding a response rate of 50.0%. Meanwhile, 80 healthy controls were enrolled. The mean age of interviewees was 45.26 ± 9.71 years, ranging from 22 to 65 years. A total of 90(56.6%) adults were female. There were no significant difference between the two groups in age, gender, marital status, BMI and years of education(*P* > 0.01). Regular tobacco use and regular alcohol intake between the two groups was different(*P* < 0.01). In the insomnia disorder group, the mean duration of symptoms was 20.92 ± 27.77, and the mean PSQI total score was 10.43 ± 3.19 (Table 1).

**Table 1:** Basic demographics of the study population

Characteristic	Insomnia(*n* = 79)	Controls (*n* = 80)	*P*
Age (year)	44.27 ± 10.22	46.24 ± 9.24	0.20
Gender Female (%)	46 (58.3%)	44 (55.0%)	0.68
BMI	23.88 ± 3.46	24.00 ± 3.13	0.82
Marital status			
Single/widower/divorced (%)	8 (10.1%)	6 (7.5%)	0.56
Married (%)	71 (89.9%)	74 (92.5%)	0.56
Years of education (year)	11.90 ± 2.73	12.09 ± 3.21	0.69
Regular tobacco use (%)	24 (30.4)	10 (12.5)	0.01
Regular alcohol intake (%)	23 (29.1)	13 (16.3)	0.06
Duration of symptoms (mo)	20.92 ± 27.77	–	–
PSQI total score	10.43 ± 3.19	–	–
Subjective sleep quality	1.87 ± 0.70	–	–
Sleep latency	2.42 ± 0.86	–	–
Sleep duration	1.44 ± 1.07	–	–
Habitual sleep efficiency	1.53 ± 1.34	–	–
Sleep disturbance	1.73 ± 0.57	–	–
Use of sleeping medications	0.66 ± 1.08	–	–
Daytime dysfunction	0.66 ± 0.68	–	–

BMI body mass index calculated as weight (kg)/height (m^2^)

### Differences of SCL-90R scores in insomnia patients and healthy controls

To assess the associations of various psychological factors with sleep development, we compared the symptomatic dimensions as well as total SCL-90R scores between patients with insomnia disorder and healthy controls. Somatization, obsessive- compulsive, interpersonal sensitivity, depression, anxiety, hostility, phobic anxiety, paranoid ideation, psychoticism, additional items and total score showed significantly higher levels in the insomnia group than in controls (all *P* < 0.01) (Table 2).

**Table 2:** SCL-90R scores in insomnia patients and healthy controls (mean ± SD)

SCL-90R	Insomnia(n = 79)	Controls (n = 80)	*t*	*P*
Somatization	1.83 ± 0.54	1.36 ± 0.26	6.989	<0.001
Obsessive- compulsive	2.15 ± 0.62	1.45 ± 0.32	8.991	<0.001
Interpersonal sensitivity	1.84 ± 0.57	1.29 ± 0.26	7.875	<0.001
Depression	1.85 ± 0.60	1.26 ± 0.19	8.296	<0.001
Anxiety	1.82 ± 0.64	1.27 ± 0.21	7.293	<0.001
Hostility	2.06 ± 0.70	1.36 ± 0.32	8.079	<0.001
Phobic anxiety	1.44 ± 0.61	1.07 ± 0.15	5.071	<0.001
Paranoid ideation	1.79 ± 0.62	1.26 ± 0.33	6.748	<0.001
Psychoticism	1.66 ± 0.50	1.22 ± 0.23	7.076	<0.001
Additional items	2.46 ± 0.51	1.27 ± 0.15	19.812	<0.001
Total score	1.86 ± 0.48	1.29 ± 0.17	10.205	<0.001

### Differences of SCSQ scores in insomnia patients and healthy controls

**Fig. 1:**
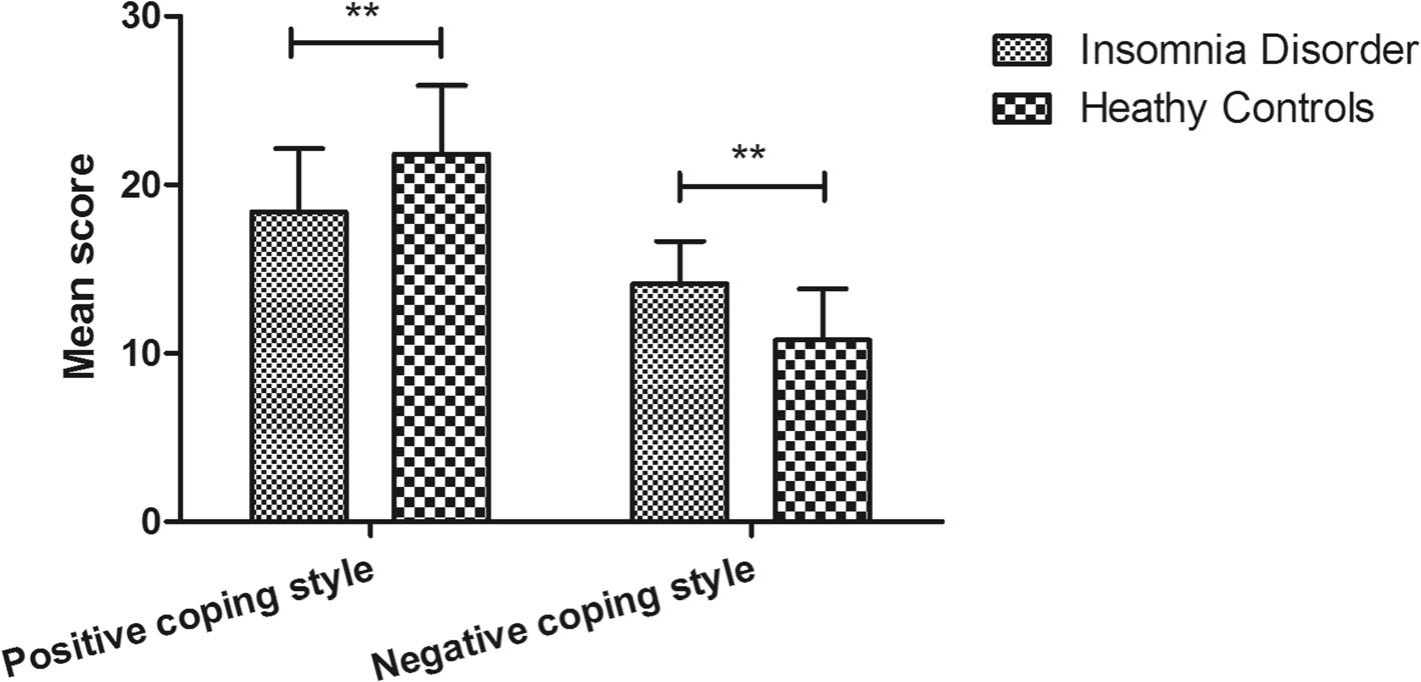
Comparison of SCSQ scores between insomnia disorder and heathy controls. **P < 0.01.T test showed that the positive coping style score in patients with insomnia disorder was significantly lower whereas the negative coping style score was higher than that in heathy controls (both *P* < 0.01)

To analyze the coping styles of insomniacs, we compared the coping styles between patients with insomnia disorder and healthy controls. The positive coping style score in the insomnia group (18.38 ± 3.78) was significantly lower than that in the control group (21.84 ± 4.05)(*P*<0.01), however, the negative coping style score of the insomnia group (14.14 ± 2.51) showed a significant higher level than the control group (10.78 ± 3.06) (*P*<0.01) Fig. 1.

### Association of coping styles, sleep disturbance and psychological distress

We next analyzed the relationship between sleep quality, psychological distress and coping styles. Correlation analyses showed that positive coping style score was adversely related to PSQI total score, sleep latency, use of sleeping medications, daytime dysfunction, SCL-90R total score, obsessive-compulsive, depression, anxiety, phobic anxiety and additional items (*P* < 0.05 or 0.01). However, negative coping style score was positively related to PSQI total score, sleep latency, Habitual sleep efficiency, somatization, interpersonal sensitivity and additional items (*P* < 0.05 or 0.01) (Table 3).

**Table 3:** Correlations of sleep quality, psychological distress and coping style in insomnia patients

	Positive coping style score		Negative coping style score	
	*r*	*P*	*r*	*P*
PSQI total score	−0.323^**^	0.004	0.346^**^	0.002
Subjective sleep quality	0.013	0.906	0.141	0.217
Sleep latency	−0.232^*^	0.040	0.295^**^	0.008
Sleep duration	−0.140	0.217	0.063	0.584
Habitual sleep efficiency	0.000	0.999	0.237^*^	0.035
Sleep disturbance	−0.137	0.229	0.151	0.183
Use of sleeping medications	−0.328^**^	0.003	0.055	0.628
Daytime dysfunction	−0.244^*^	0.030	0.009	0.935
SCL-90R total score	−0.247^*^	0.028	0.213	0.059
Somatization	−0.114	0.315	0.249^*^	0.027
Obsessive-compulsive	−0.251^*^	0.025	0.104	0.361
Interpersonal sensitivity	−0.202	0.074	0.241^*^	0.032
Depression	−0.252^*^	0.025	0.147	0.197
Anxiety	−0.226^*^	0.045	0.207	0.067
Hostility	−0.113	0.321	0.037	0.743
Phobic anxiety	−0.254^*^	0.024	0.054	0.636
Paranoid ideation	−0.128	0.262	0.138	0.225
Psychoticism	−0.152	0.182	0.205	0.069
Additional items	−0.358^**^	0.001	0.273^*^	0.015

*P<0.05, ** *P*<0.01

### Independent influencing factors of sleep quality

Multiple stepwise regression analysis was used to investigate the independent influencing factors of sleep quality (PSQI total scores). The initial independent variables included gender, age, BMI, years of education, duration of symptoms, ten symptom dimensions of SCL-90R, positive coping style and negative coping style. In insomnia patients, PSQI total score was independently and positively correlated with additional items of SCL-90R (standardized β = 0.856, *P* < 0.01) and negative coping style score(standardized *β* = 0.113, *P* < 0.05) (Table 4).

**Table 4:** Multiple stepwise regression analysis showing the variables independently associated with sleep quality

	Standardized *β*	*t*	*P*
Additional items of SCL-90	0.856	15.962	<0.001
Negative coping style score	0.113	2.099	0.039

## Discussion

In this study,we explored the the coping styles and psychological distress among patients with insomnia. In this study, positive coping style score was lower and negative coping style score was higher in insomnia group than in control group. The results support the hypothesis that insomniacs are more likely to use negative coping style, whereas less likely to use positive coping style. Coping can be distinguished into emotion-focused coping, problem-focused coping, and avoidance-focused coping [30, 31]. Commonly, problem-focused coping is associated with better consequences and is therefore regarded as positive coping. However, emotion-focused coping and avoidance-focused coping are associated with poor outcomes and are conceived of as negative coping [31]. One prospective study showed that undergraduates who typically used a positive coping style to deal with stress had longer sleep, whereas those who used a negative coping style had shorter sleep [17]. Recently one Japanese study demonstrated that the negative coping styles “avoidance and suppression” were significantly correlated with current insomnia in patients with type 2 diabetes mellitus [19]. This study found increased use of negative coping style in the insomnia population, which was consistent with previous research in other population with poor sleep quality. These findings may prompt clinicians to focus on the coping styles when confronting stress in patients with insomnia.

This study found that all dimensions of SCL-90R in patients with insomnia disorder were higher than normal controls, such as somatization, obsessive-compulsive, depression, anxiety and hostility. Pallesen and his colleagues [32] using SCL-90R in eldly insomniacs showed higher scores of somatization, interpersonal sensitivity, obsessive-compulsive, depression, anxiety, hostility and phobic anxiety than good sleepers, which was almost in line with our study. These results demonstrated that all aspects of the psychology of insomniacs are adversely affected. Previous studies have shown that the risk of developing depression in the future was associated with insomnia at baseline, and insomnia treatment can prevent incidence of depression symptoms in those with insomnia [33–35]. This study showed a significant increase in depression score of insomniacs, which indicate we should pay attention to depression associated with insomnia and avoid the aggravation of depressive symptom.

In the present study, positive coping was adversely associated with insomnia symptoms, whereas negative coping was positively related to insomnia symptoms. In addition, the study also found the correlation between coping styles and psychological symptoms. The results support another hypothesis that coping styles significantly associate with the severity of insomnia and the psychological status. Previous studies have demonstrated that positive coping style predicted better sleep, whereas negative coping style was related to sleep disturbance in other physical disease with insomnia symptom [20–22], which was consistent with this study in patients with insomnia disorder. Even several earlier studies have shown that coping styles mediate between stress and anxiety, depression, and sleep [17, 31, 36]. Therefore, doctors should place more emphasis on the relationships of coping styles and clinical symptoms in order to maximize the benefit of patients with insomnia disorder. Contrary to study hypothesis and previous research, another study considered coping styles were not linked with insomnia symptom among those with stress-related insomnia [18]. The discrepancy could be explained by difference in other control factors such as environment and anxiety. Further multiple stepwise regression analysis in our study found that negative coping style was an important contributor to sleep quality in patients with insomnia disorder. Identifying the negative coping style that strongly associated with insomnia symptoms in insomniacs, is imperative to develop more specific interventions to coping styles.

There are several limitations in this study. For example, this study used self-administered questionnaires to measure sleep quality, coping styles and psychology distress. Objective inspection methods such as polysomnography to examine sleep are needed in future research. Besides, this was a cross-sectional study in which the relationship between coping styles, insomnia disorder and psychological distress could not be determined in terms of temporal trends and causal relationships. A future longitudinal study is needed to examine how coping styles influence sleep quality in patients with insomnia disorder.

## Conclusions

In conclusion, this study demonstrated that psychological distress is more obvious in patients with insomnia disorder than heathy controls. Insomniacs use more negative coping styles and less positive ones. Positive coping is adversely associated with insomnia symptoms and psychological distress, whereas negative coping is positively related to those symptoms. In insomnia disorder, negative coping style is identified among the most significant factors affecting sleep quality. Therefore, personalized psychological treatments aimed at reducing negative coping style should be applied to improve insomnia symptoms.

## Data Availability

The datasets used and/or analysed during the current study are available from the corresponding author on reasonable request.

## Abbreviations

PSQI: Pittsburgh Sleep Quality Index
SCL-90R: Symptom Checklist-90-Revised
SCSQ: Simplified Coping Style Questionnaire
DSM-5: Diagnostic and Statistical Manual of Mental Disorders, Fifth Edition
BMI: Body mass index
SD: Standard deviation
